# Response Time Reduction Due to Retesting in Mental Speed Tests: A Meta-Analysis

**DOI:** 10.3390/jintelligence6010006

**Published:** 2018-02-26

**Authors:** Jana Scharfen, Diego Blum, Heinz Holling

**Affiliations:** Institute of Psychology, Westfälische Wilhelms-Universität Münster, 48149 Münster, Germany; blumworx@gmail.com (D.B.); holling@uni-muenster.de (H.H.)

**Keywords:** meta-analysis, mental speed, processing speed, retest effect, practice effect, response time, reaction time, automatization

## Abstract

As retest effects in cognitive ability tests have been investigated by various primary and meta-analytic studies, most studies from this area focus on score gains as a result of retesting. To the best of our knowledge, no meta-analytic study has been reported that provides sizable estimates of response time (RT) reductions due to retesting. This multilevel meta-analysis focuses on mental speed tasks, for which outcome measures often consist of RTs. The size of RT reduction due to retesting in mental speed tasks for up to four test administrations was analyzed based on 36 studies including 49 samples and 212 outcomes for a total sample size of 21,810. Significant RT reductions were found, which increased with the number of test administrations, without reaching a plateau. Larger RT reductions were observed in more complex mental speed tasks compared to simple ones, whereas age and test-retest interval mostly did not moderate the size of the effect. Although a high heterogeneity of effects exists, retest effects were shown to occur for mental speed tasks regarding RT outcomes and should thus be more thoroughly accounted for in applied and research settings.

## 1. Introduction

Requesting examinees to complete the same mental speed test more than once is common both in applied and research settings. For a clinical example, assessing cognitive decline in a patient may require the administration of one mental speed test at two different points in time. Also, in organizational selection settings, mental speed tests are common tools to assess vocational aptitude [1]. As most applicants prepare for selection settings, retest effects might influence their test results [2]. In randomized controlled trials evaluating intervention effectiveness by pre- and post-measurements, persons are retested with mental speed tests as criterion tasks [3]. Further, research on cognitive development often has to take into account retest effects, because, in longitudinal studies, retest effects might contaminate the measurement of cognitive abilities [4].

The size of score gains due to retest effects in a broad range of cognitive ability tests has been meta-analyzed by Kulik, Kulik and Bangert (1984) [5], Hausknecht et al. (2007) [2], Calamia et al. (2012) [6] and Scharfen, Peters and Holling (in press) [7]. These authors found retest effects from the first to second test of 0.23 to 0.42 standards deviations (*SDs*), with effect sizes depending on several moderators, such as the test-retest interval, equivalence of test forms (i.e., whether alternate or identical) and sample characteristics like age and general mental ability. Further moderating variables were suggested by Randall & Villado (2017) [8].

Although both speed and accuracy are known to have a direct impact on cognitive ability test performance [9,10], meta-analytical and most primary research on retest effects has been focusing solely on score gains, or on improvements in accuracy and neglecting improvements regarding response time (RT) due to retesting. To the best of our knowledge, no meta-analysis has been reported that summarizes retest effects concerning the time needed to complete a task, defined as RT reductions due to retesting. Thus, it is the goal of this meta-analytic review to provide results of retest effects on RT reductions in mental speed tasks, to give estimates of the size of the effect and to investigate possible moderating variables. Further, the high number of test administrations for which RT reductions and their moderators are meta-analyzed are of special interest, as this study contributes to a better understanding of the longitudinal proceeding of retest effects over several test administrations. This meta-analysis stresses that mental speed tasks and RT outcomes are not resistant to retest effects and should be considered in all applied settings.

### 1.1. Retest Effects in Cognitive Ability Tests

The repeated administration of cognitive ability tests results in so-called retest effects [11], practice effects [2] or testing effects [12]. Retest effects describe the gain in test performance that results from the repeated administration of the same or an alternate but equally difficult cognitive ability test to the same sample [11,13]. Evidence for the existence of retest effects is vast [2,5,6,7].

Three groups of causes of retest effects have been summarized by Lievens, Reeve and Heggestad (2007) [11]: ability increase (i.e., a true change in the latent construct), contamination reduction (i.e., less influence of construct-irrelevant variables) and increase of test-specific skills and strategies. The first cause, which considers participants’ latent cognitive ability to increase because they are retested, can also be referred to as the testing effect that describes learning processing due to testing [12,14,15]. This cause, latent change due to retesting, has to be differentiated from latent change due to cognitive development. When considering that cognitive abilities develop throughout the lifespan [4,16], test results from two different time points can reflect a true change in the latent construct that is due to developmental change and less due to retesting. This is especially probable when retested within a long test-retest interval or during a period in life where cognitive abilities change comparably fast (i.e., during childhood and higher age). In fact, Finkel et al. (2007) [16] found that developmental changes in mental speed can explain developmental changes in fluid intelligence. The first cause explaining why retest effects evolve does not refer to cognitive development but implies that the latent cognitive ability measured by the criterion test is improved due to retesting [11]. Thus, if the first cause is assumed to be accountable for retest effects, cognitive development should be excluded to be responsible for latent change within the test-retest interval or population under observation. As validity changes have often been observed as a consequence of retesting [2,17,18,19], it seems rather implausible that latent change is responsible for retest effects unrestrictedly.

The second group of causes, focusing on construct-irrelevant variables such as situational test anxiety, motivation and familiarity, gained more empirical support. For example, Matton, Vautier and Raufaste (2009) [20], Freund & Holling (2011) [21], Reeve & Lam (2007) [13], Reeve, Heggestad and Lievens (2009) [22] found evidence for these factors influencing retest performance. Also for the third groups of causes, evidence for test-specific strategies and skills has been put forward by a number of studies investigating strategy use of the efficacy of strategy-induction by, e.g., coaching programs [2,20,21,22,23,24,25,26].

### 1.2. Mental Speed

Many different concepts associated with mental speed can be found in the literature, such as speed of information processing or perceptual speed. But the term mental speed can be reserved to designate “the human ability to carry out mental processes, required for the solution of a cognitive task, at variable rates or increments of time” [27] (p. 29). Specifically speaking, mental speed can be defined as a cognitive ability that consists of quickly encoding, transforming and retrieving information, as well as a measure of attention, working speed and ease of perception [28,29]. “The faster the rate of processing, the greater the amount of information that can be processed in one unit of time” [30] (p. 165). It can be considered a constituent part of general intelligence and was found to be related to other facets of intelligence such as reasoning ability [31,32,33].

Generally, both accuracy and speed play a central role in test performance [9,10]. Mental speed tests most commonly either establish a certain time limit to complete a high number of tasks, or measure RT that is needed to complete a task. During the last decades and as a result of improved feasibility of RT measurement due to digitalization and computerization, reporting RTs has become more frequent [9]. Actually, two major indicators of performance apply: Firstly, accuracy, as reflected in the number of items completed correctly or appropriate solutions derived during a given time span and other related score measures. RT, secondly, indicates the time spent to respond to an item or to give a solution, independent from its correctness. Ideally, a mental speed item is solved both quickly and correctly, as accuracy and RT maintain a reciprocal relation overall but mean and variability are not always preserved when transforming one of these measure types to the other [9,34]. In fact, the concept of speed-accuracy tradeoffs implies that spending more time solving an item raises the amount and quality of information to be processed and, as a consequence, improves response accuracy [35]. On the contrary, there is evidence for independence of accuracy and RTs, or at least for a more complex relationship that might be moderated by, e.g., task complexity and participants’ general mental ability [9,36,37,38]. In other words, score gains and RTs appear not to be exchangeable variables; thus, results from each of these two outcomes may be interpreted differently. Hence, it might give new insights to evaluate retest effects with reference to RTs besides those regarding score gains.

According to Villado, Randall and Zimmer (2016) [39], retest effects can be more or less critical depending on the construct of interest as well as certain test characteristics (e.g., heterogeneous vs. homogeneous item types). Mental speed, as one construct of interest that should be prone to retest effects and that is often measured by homogeneous item types and by RT outcomes, has only rarely been focused by retesting studies. Therefore, this meta-analysis contributes to the field by explicitly focusing the construct of mental speed and RTs as outcomes.

### 1.3. RT Reduction Due to Retesting in Mental Speed Tasks

The three groups of causes that can lead to retest effects according to Lievens et al. (2007) [11] have been explained above. It becomes evident that a reduction of RTs parallel to score gain increase due to retesting would be expected. Indeed, older developmental research from Baltes, Dittmann-Kohli and Kliegl (1986) [40] found that both accuracy and speed in several cognitive ability tasks can be fostered by retesting. Firstly, if the latent ability is improved due to retesting, this would be reflected in shorter RTs in mental speed tasks. Secondly, a reduction of construct-irrelevant factors, such as situational test anxiety or unfamiliarity with the test, would lead to a reduction of RTs as well. Lastly, if strategies and skills are employed in repeated tests, this would also lead to shorter RTs. To sum up, a reduction of RTs due to retesting is suggested when referring to any of the three groups of causes of retest effects [11].

In 1987, Ackerman [34] established a difference between how testees typically perform when a novel test is presented to them and how they perform in the context of repeated test administration, while differentiating between score and RT changes. From this point of view, when repeating a task, automatization of mental processes takes place progressively over controlled mental processes [41,42,43,44,45,46]. Different abilities that underlie mental speed have a different impact on performance at early and later stages of automatization: When working on a test for the first time, reaction times might resemble perceptual speed ability, whereas after practicing multiple times, psychomotor ability would be reflected in RTs [1,34]. The automatization of a skill leads to less effort, less deterioration under stress and, most importantly, a much faster response [34,47]. In other words, RT is expected to decrease across test repetitions [1]. Automatization and the three groups of causes are assumed to have a reciprocal positive influence on each other. For example, automatization might lead to less test anxiety and at the same time less test anxiety might also foster automatization.

According to the power law of practice [48,49,50,51], the benefits from practice follow a non-linear function, where improvements are rapid at first but then decrease towards more practice. Given this idea, RTs would decrease most in the first tests, followed by decreasing RT reductions in further tests.

However, retest effects with RTs as outcome measures have been given less attention than those concerning score gains. A few primary studies directly address the question of the size of decrease in RTs as a consequence of retaking a test [1,52,53,54,55,56,57,58]. Wöstmann et al. (2013) [58] administered cognitive ability tests twice to a sample of 23 healthy participants, with four variables of the Eriksen flanker, the Simon and the Stroop tasks showing reduced variability of RTs, as well as an RT improvement for incongruent trials at the second administration. Soldan, Clarke, Colleran and Kuras (2012) [56] repeatedly administered perceptual classification tasks comprising unfamiliar objects to a group of 48 testees. Results suggested RT improvements as well as higher accuracy when the same response was required during the encoding and test phases across trials. In a study by Hagemeister (2007) [1], 30 participants were assessed with an attention test at the beginning as well as at the end of the experiment. A mean RT decrease throughout the study was observed. Findings supported the power law of practice [48,49,50] being applicable when retesting with mental speed tasks: Retest effects between consecutive administrations decreased with the number of test repetitions. Rockstroh and Schweizer (2004) [54] examined results from RT tasks on 83 males and RTs were significantly shortened across retest-practice sessions as well. Collie, Maruff and Darby (2003) [53] assessed 113 individuals with automated cognitive tests in four administrations over the course of one day and found RT reductions that diminished towards the latest trial, again reinforcing the assumption of decreasing gains. All in all, RT improvements are shown to be a result of retesting mental speed over several studies with different test-retest intervals and research settings. Also, there is evidence that RT reduction over several test repetitions might decrease with the number of test administrations [1,53].

Although not many studies investigated RT improvements between test administrations, the more employed research field of serial learning often employ RTs as measures for learning effects. Serial learning refers to within-test retest effects over subsequent trials. Within-test retest effects in RT seem to be a stable result from this field [59,60,61]. Though, the focus of the current meta-analysis lies on between-test retest effects regarding RTs.

To summarize, there is high theoretical and empirical support to expect retest effects in RTs and mental speed tests. Thus, the following hypothesis is derived: RTs decrease with the number of test administrations, with the largest RT reduction from first to second test administration (H1).

### 1.4. Moderators of RT Reduction Due to Retesting in Mental Speed Tasks

Retest effects with reference to score outcomes can be affected by several moderators, thus supposedly impacting RT reduction as well [2,6,8]. In this meta-analysis, we focus on those moderators that were most commonly reported by eligible studies and could be coded reliably.

#### 1.4.1. Test Form

Score gains are usually larger when identical test forms are administered during retesting compared to alternate but equally difficult forms of a test [2,5,7,21,24]. In this context, score gains can be explained by memory effects that exist only for identical test forms [62]. Recognizing an identical item can lead to remembering the answer to this item but also to a decreased test anxiety, a higher familiarity and a more feasible application of test-specific strategies. Besides higher scores, recognizing identical items can also lead to a faster response, which means less time is spent on familiarizing with the item and the solving process. Therefore, less RT reduction should take place when alternate test forms are administered.

The concept of automatization is definite to consistent stimuli. Thus, performance in alternate tasks would lead to automatized processes less, because stimuli are less consistent and therefore to longer RTs [45,46]. Thus, hypothesis 2 (H2) proposes that RT improvements are larger when identical compared to alternate test forms are administered in further test administrations [45,46].

#### 1.4.2. Task Complexity

Task complexity can be determined by the number of basic cognitive operations involved in solving a task. It has been claimed to moderate retest effects in several studies and, over several test repetitions, tasks with higher complexity were mostly found to show larger retest effects [7,8,63]. It is argued that tasks that are more complex are more prone to retest effects. In easy tasks, only few construct-irrelevant factors can be reduced. For example, in simple tasks, rule comprehension should be already fully achieved within the first test and no further reduction of incomprehension is possible. Also, a higher number of test-specific skills and strategies could be developed in more complex tasks [7]. The studies mentioned here focus on a broad range of cognitive abilities. However, as factors like construct-irrelevant factors and test-specific strategies are assumed to cause RT reductions in mental speed tasks as well, these mechanisms might apply for a differentiation within mental speed tasks and for RT outcomes.

Ackerman 1987 [34] argues that automatization is facilitated in consistent tasks and that performance can be improved rapidly in these kinds of items. For inconsistent tasks, he argues, controlled processing plays a more important role and sets the limits for maximum performance on the task. Simple tasks, in which a simple RT to a stimulus is measured, are indeed more consistent compared to complex tasks, in which additional cognitive abilities are required. For simple tasks, automatization might thus take place very quickly, maybe even within the first test session. In more complex tasks, it might take longer for automatization to develop and RT reductions might still be observed in further test administrations. Thus, it is hypothesized (H3) that larger RT reductions will be observed in tasks with higher complexity.

#### 1.4.3. Test-Retest Interval

Calamia et al. (2012) [6], Hausknecht et al. (2007) [2], Scharfen et al. (in press) [7] and Salthouse et al. (2004) [64] observed a moderating effect of the length of the test-retest interval between administrations on the size of the score gains due to retesting. The influence of memory effects is argued to be directly related to the length of the test-retest interval because memory decreases over time. The longer the test-retest interval, the less information about the test can be recalled. Thus, less test-specific strategies and skills would be recalled and the initial reduction of construct-irrelevant factors might have revoked when retesting after a long time interval, which is assumed to lead to less RT reduction. It is also plausible to assume that automatization declines with the length of the test-retest interval. Accordingly, Hypothesis 4 (H4) predicts lower RT reductions resulting from longer test-retest intervals. In addition, Hypothesis 4a (H4a) suggests an interaction of test-retest interval and test form: Test-retest interval might have a larger influence on retest effects in identical test forms compared to alternate test forms.

#### 1.4.4. Age

The impact of sample characteristics such as age on the estimated RT change due to retesting has been studied by, e.g., Calamia et al. (2012) [6], Bürki, Ludwig, Chicherio and Ribaupierre (2014) [3], Howard et al. (2004) [65], Van Iddekinge et al. (2011) [66], Verhaeghen (2015) [4] and Scharfen et al. (in press) [7]. Evidence for RT reductions depending on age is mixed. For example, Bürki et al.’s (2014) [3] research conducting a 10-day working memory training in 63 younger and 65 older adults showed that both age groups exhibited similar training gains over the course of training. RTs decreased similarly, as well as accuracy, although RTs were generally greater in the older group. On the other hand, Howard et al. (2004) [65] observed differences in RT improvements in sequence learning tasks between younger and older age groups. Calamia et al. (2012) [6] found a significant influence of age on score gains due to retesting, suggesting that the estimated retest score gain is slightly reduced with age.

From a theoretical point of view, fluid intelligence and the ability to maintain information tend to decrease with age [67,68]. This might affect the ability to learn from prior test experience [58]. Also, the ability of automatizing task performance is observed to decline with age [69,70]. It is therefore hypothesized that a higher age will be associated with lower RT reductions (H5).

## 2. Methods

### 2.1. Inclusion and Exclusion Criteria

The following criteria were considered for inclusion and exclusion of studies: According to the definition of retest effects [11,13]; (a) the same or an alternate but equally difficult version of a mental speed test had to be administered at least twice within the same sample under equal conditions. We included cognitive ability tests that could be defined as measuring (b) mental speed according to the Berlin Intelligence Structure Model (BIS) [28,29]. (c) RTs and their standard deviations (*SDs*) had to be reported for at least two test administrations, or effect sizes had to be given. (d) The mean age of the samples had to be between 12 and 70 years in order to prevent developmental latent changes in cognitive abilities to account for changes between measurements. As mentioned above, in childhood and higher age, latent cognitive change is more probable to occur [67,71,72]. We excluded these age groups in order to be able to exclude developmental change as a main reason for RT change between tests and to facilitate a consistent interpretation of the effect and the factors causing it. Also, samples had to be (e) without clinical disadvantages such as any type of illness or clinical syndrome, as retest effect can be expected to be smaller in clinical samples [6]. (f) Only studies written in the English or German language were included.

### 2.2. Literature Search

**Figure 1:**
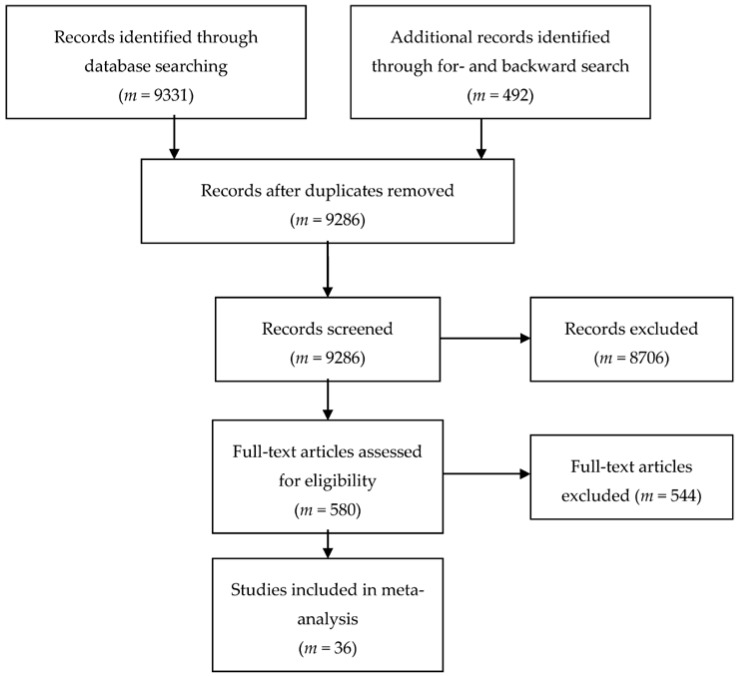
Flow chart of the literature search and study selection process according to Moher, Liberati, Tetzlaff and Altman (2009) [89].

The literature search and study selection process is depicted in Figure 1. A keyword online search was conducted for studies published between January 1990 and April 2017. A combination of the terms test*, assess*, cognit*, intelligen*, “aptitude test,” “achievement test,” “IQ,” processing speed,” “mental speed,” retest*, repeat*, repetit*, practice, retak*, train*, coach*, fast*, “reaction time,” RT, speed*, quick*, pace, accelerat* and rapid was used searching the databases PsycARTICLES, PsychINFO and PSYNDEX in joint basis. The operator asterisk (*) indicates that different word endings were taken into account. This search yielded 9331 articles that were screened for eligibility. Test manuals were checked for eligibility as well but relevant test-retest data in RT outcome format was not reported.

Additionally, we performed a forward and backward search based on relevant meta-analytic reviews evaluating either retest effects or training and coaching interventions of cognitive ability [2,5,6,7,73,74,75,76,77,78,79,80,81,82,83,84,85,86,87,88].

After removing duplicates, 9286 articles were screened, of which 580 were assessed for eligibility in detail. Finally, *m* = 36 studies including *k* = 49 samples and *o* = 212 outcomes were identified as fitting the inclusion and exclusion criteria.

### 2.3. Coding

A coding scheme was developed that included study, sample and test characteristics, information on relevant moderators and RT outcomes (*Ms*, *SDs*). All studies were coded by one of the authors. Coding was carefully double-checked to prevent coding errors. For this meta-analysis, it was not necessary to assess risk of bias (e.g., [89]), because strict inclusion criteria limited the study design to a simple retesting setting in which criteria of risk of bias do not apply. This meta-analytic study had not been pre-registered.

Task complexity was coded dichotomously; tasks were coded as simple if they asked for a basic reaction to stimuli without making a choice, differentiation and without inhibition of other stimuli being required. Tasks were coded as complex if the task requested any additional ability, such as ordering or comparing stimuli, or inhibiting reaction to other stimuli. See Supplementary File “data.R” for the complete coding scheme and coding of all studies.

### 2.4. Effect Size Calculation

Effect sizes were calculated for up to four test administrations using the function escalc() and the metafor package [90] for *R* [91]. RTs were compared between two test administrations within the same samples. Thus, the *standardized mean change* was chosen as the effect size and standardized by raw score standardization (*SMCR*) [90,92,93,94] for each comparison c between administrations as follows:

(1) $$SMCR_{c}=\frac{M_{t}-M_{1}}{SD_{1}},$$

where *M*_1_ is the mean RT of the first test, *SD*_1_ is its standard deviation and *M*_t_ is the mean RT of the respective test repetition *t*: second, third or fourth test.

The sampling variance of *SMCR* is calculated as

(2) $$Var(SMCR_{c})=\frac{2\times \left(1-r_{c}\right)}{n_{t}}+\frac{SMCR_{c}^{2}}{2\times n_{t}}$$

where *r*_c_ is the correlation between outcomes of the first and the respective *t* administration and *n*_t_ is the sample size at the respective *t* administration. Note that the sampling variance corresponds to the squared standard error. Eligible studies reported *r*_c_ for only 16.04% of the outcomes and, hence, it had to be estimated for those studies that did not report it. Assuming smaller correlations between reaction times with an increasing test retest-interval [95], *r*_c_ were predicted by a linear model using the test-retest interval as a predictor. Resulting estimates of *r*_c_ had a mean of *M* = 0.64 (*SD* = 0.14). Sensitivity analyses were performed in order to control for variability of results due to the choice of *r*_c_. 

### 2.5. Meta-Analytic Strategy

As studies often reported results from multiple samples and also multiple outcomes per sample, a multilevel meta-analysis was conducted, modelling outcomes as nested in samples and samples nested in studies. In addition, a variable was included indicating the comparison that the effect size refers to (comparison = 1.2, 1.3, 1.4) [96]. This variable was again modeled as nested in outcomes. Thus, we analyzed the value of τ^2^_c_ per comparison, which indicates the total amount of variation among effects observed for the different levels and it is therefore a measure of overall heterogeneity of the true effect. Further, contrast variables (t2, t3, t4) were added in order to test differences between test administrations by one comprehensive meta-analysis. The following meta-analytic model was used:

*y_ijc_* = *μ_c_* + *u_ic_* + *w_ijc_* + *ε_ijc_*, (3)

where, for each comparison of administrations c, *y_ijc_* is the *j*th effect size from the *i*th study, *μ_c_* is the true SMCR, *u_ic_* is a random effect at the level of studies, *w_ijc_* is a random effect at the level of samples and *ε_ijc_* is the sampling error [97].

In longitudinal meta-analyses comparing several test administrations as the current one, correlations between outcomes are often assumed to show autoregressive or heteroscedastic structures [90,98,99,100]. Thus, four different models were tested against each other, each specifying a different variance-covariance structure between random effects (i.e., comparisons). The first model assumed a compound symmetric structure, meaning the variance of random effects was assumed to be equal for all comparisons. The second model allowed an unstructured variance-covariance matrix, resulting in independent variances for each comparison. The third model assumes homoscedastic autocorrelations between comparisons. The last model allowed heteroscedastic autocorrelations between comparisons. For further details, see Viechtbauer (2010) [90]. For this analysis, the last model assuming different amounts of heterogeneity and autocorrelations between random effects showed the best fit (*p* < 0.001), which is why results are reported on the basis of this model.

Multilevel modeling does not fully account for dependencies between comparisons, outcomes, samples and studies regarding estimation of standard errors. Thus, robust variance estimation had to be applied in order to achieve reliable standard errors [101]. Studies were used as clusters.

Moderators were tested by including corresponding variables into the model. Contrasts were specified by linear hypotheses to test differences between categories and comparisons. Note than when analyzing RT reductions between subsequent test administrations (i.e., 2.3 and 3.4), linear hypotheses test the following differences: 2.3 = 1.2 vs. 1.3 and 3.4 = 1.3 vs. 1.4. They can thus be interpreted as RT reductions between subsequent tests but also as the differences between two RT reductions that compare a different administration to the first one. Associations between moderators were calculated in order to be aware of possible confounding of effects. One-sided *p*-values are reported for directed hypotheses and an α level of 0.05 was applied. A funnel plot based on residual values from the main model without moderators plotted against their standard errors were inspected for publication bias. A funnel plot based on the residual values from the main model was chosen, because only one plot is required that allows for inspection of publication bias for the overall meta-analysis including contrast variables for comparisons. For complex multilevel models like the current one, appropriate methods to judge publication bias quantitatively have not yet been derived. See Supplementary File “script.R” for analyses.

## 3. Results

### 3.1. Study, Sample and Test Characteristics

The main study, sample and test characteristics are summarized in Table 1. See Table A1 in the Appendix A for a full list of eligible studies, their main characteristics and effect sizes.

Studies were published between 2010 and 2016. All of the studies included in the meta-analysis had been published in peer-reviewed journals and conducted in experimental settings. Sample information is given for the total sample and thus weighted by sample sizes. Note that one study [102] had a very high sample size of *n* = 19,330, which explains the high *SD* of the total sample size *N*. Tests used most frequently were the Trail Making Tests (TMT) [103] or similar versions of this paradigm (*o* = 42, 19.81%) and Stroop tasks [104,105] (*o* = 22, 10.38%). Note that *SMCR_c_* is the unweighted average of all observed effect sizes, whereas when presenting meta-analytic results, the meta-analytically weighted average of *SMCR_c_* as an estimate for *μ_c_* is reported.

### 3.2. RT Reduction

Results from the main analysis without including moderators can be found in Table 2. See Supplementary File 1 for a forest plot including all studies and effect sizes.

H1 was supported as significant RT reductions were observed for the first test repetition (*SMCR*_1.2_ = −0.237) and for later ones (*SMCR*_1.3_ = −0.367, *SMCR*_1.4_ = −0.499). RT reductions from first to second repetition were significantly smaller compared to those comparison first to later comparisons and RT reductions from first to third test were smaller than those from first to fourth test, as indicated by *SMCR*_2.3_ = −0.131 (*p* = 0.021) and *SMCR*_3.4_ = −0.132 (*p* < 0.001).

RT reduction increased with the number of tests. When comparing the change from first to second test, this decrease was significant (*SMCR*_1.2 vs. 2.3_ = −0.106, *p* = 0.041), indicating that the largest retest effect between subsequent administrations was observed from first to second test. Effect sizes did not differ significantly when comparing later subsequent administrations (*SMCR*_2.3 vs. 3.4_ = 0.002, *p* = 0.979). It seems that a plateau has not been reached after four test administrations, because RTs still show significant reductions between administrations that are comparably large.

The estimated overall standard deviations of the true effect τ reached very high values for all comparisons. These estimates stress a high heterogeneity of effects that will be discussed below.

Sensitivity analyses indicated that the choice of *r*_c_ did not substantially influence the size of the effects. When setting *r_c_* to 0.30 and 0.90, main results varied within the range of *SMCR*_1.2_ = [−0.243, −0.230], *SMCR*_1.3_ = [−0.379, −0.361] and *SMCR*_1.4_ = [−0.510, −0.499]. Results did not change substantially when Lyall et al. (2016) [102], who had a high sample size of n = 19,330, was excluded.

Stroop and TMT tasks can be considered two of the most commonly employed mental speed tasks and it might therefore be especially relevant to be able to estimate retest effects for these specific tests when utilizing them in assessments. As a high proportion of mental speed tests included in the meta-analysis were either Stroop tasks or variants of the TMT, results for these tests are given separately in Table 3. For more than two test administrations, less than five outcomes were observed which is why results are reported for up to three test administrations. Effect sizes did not differ significantly between the two kinds of tests.

### 3.3. Moderators

It was not possible to test H2 and H4a because of the low number of outcomes using alternate test forms (*o* = 4, 3.18%). Results for the subgroup analyses regarding task complexity can be found in Table 4. For test-retest interval and age, results of meta-regressions are presented in Table 5.

Significantly larger RT reductions were observed in complex compared to simple tasks, supporting H3. This difference between simple and complex tasks became larger comparing first to second and first to third test (∆*SMCR*_1.2_ = 0.159, *p* = 0.001, ∆*SMCR*_1.3_ = 0.295, *p* = 0.004, ∆*SMCR*_2.3_ = 0.135, *p* = 0.029). Task complexity as a moderator explained 11.7% of the overall random effect variance.

Note that for simple tasks, SMCR_1.3_ and SMCR_2.3_ were not significantly larger than zero (*SMCR*_1.3_ = −0.130, *p* = 0.071, *SMCR*_2.3_ = −0.022, *p* = 0.645). However, for simple tasks, RT reductions did neither differ significantly from first to second compared to first to third test (*SMCR*_2.3_ = −0.022, *p* = 0.135), nor did they differ significantly between first to second and second to third test (*SMCR*_1.2 vs. 2.3_ = −0.087, *p* = 0.152). Thus, comparably large RT reductions were found for all comparisons, suggesting a plateau being reached already after the second test administration.

In contrast, for complex tasks, RT reductions were significantly different from zero for all comparisons (*SMCR*_1.2_ = −0.268, *p* = < 0.001, *SMCR*_1.3_ = −0.425, *p* = < 0.001) and effect sizes referred to the first test administrations increased significantly over test repetitions (*SMCR*_2.3_ = −0.157, *p* = 0.028). A plateau seems not yet being reached, as indicated by similar subsequent RT reductions between first to second and second to third test (*SMCR*_1.2 vs. 2.3_ = −0.157, *p* = 0.122). For more than three tests, less than ten outcomes were observed for simple tasks.

RT reductions were moderated by test-retest interval when comparing first to second test administrations (*b*_1.2_ = 0.001, *p* = 0.038). RT reductions decrease with increasing time intervals between the first and second administrations. To illustrate, a test-retest interval of 1 year would lead to a predicted RT reduction that is smaller by 0.001 × 52 = 0.052 *SDs* when compared to an immediate retest. This can be considered a small effect. For further comparisons, test-retest interval did not have a significant influence on RT reductions. H4 was supported only partly.

Age moderated RT reductions between the third and fourth test (*b*_3.4_ = 0.002, *p* = 0.004). With increasing age, RT reductions between the third and fourth test thus become smaller by 0.02 *SDs* per age decade. This can be considered a small effect. Participant age had no moderating influence for any of the other comparisons between test administrations. Hence, H5 was supported only partly.

The correlation between age and test-retest interval was small and not significant (*r* = 0.07, *p* = 0.309). Task complexity was not significantly associated neither with age (*r*_complexity.age_ = −0.125, *p* = 0.068)) nor with test-retest interval (*r*_complexity.interval_ = 0.017, *p* = 0.807). All correlations are (pointbiserial) Pearson’s correlation coefficients and were tested against zero. Confounding between moderators can therefore be excluded.

### 3.4. Publication Bias

**Figure 2:**
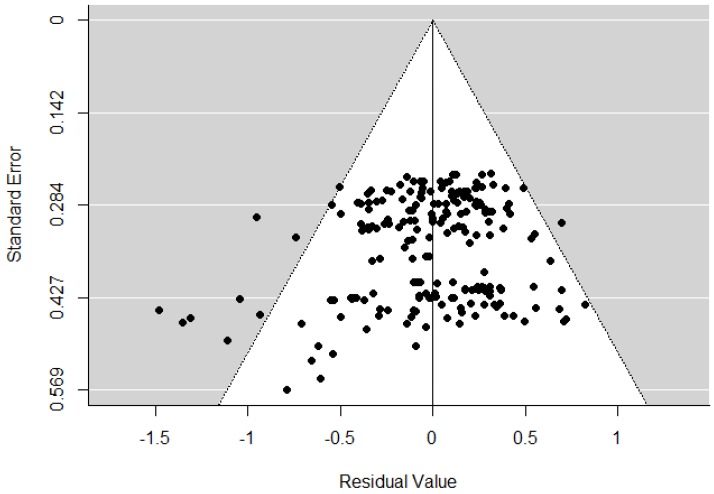
Funnel plot for inspection of publication bias. Residual values from the model without moderators are plotted against their standard errors.

A funnel plot is shown in Figure 2. In this plot, the residual values resulting from the model without moderators are plotted on the *x*-axis, along with their standard errors on the *y*-axis [106]. Publication bias can be critical if only few studies with small sample sizes report small effects. In the case of asymmetry of a funnel plot, publication bias can thus be an issue. In this meta-analytic study, no severe asymmetry becomes obvious from the plot. Publication bias does thus not seem to be crucial.

## 4. Discussion

The goal of this meta-analysis was to analyze retest effects in mental speed tests for RT measures, give estimates of the size of RT reduction due to retesting and to identify possible moderators of this effect. As retest effects are most commonly defined as score gains due to retesting with the same or a parallel version of a test, this study analyzes the effect from a different point of view by focusing on RTs. For mental speed tasks, our results show that RTs are reduced due to retesting. RT reductions were largest from the first to second test and remain significant over several test repetitions. Test-retest interval and age partly moderate the size of this effect, whereas smaller RT reductions were observed in simple compared to more complex tasks.

### 4.1. Summary of Results and Theoretical Implications

Retesting with mental speed tasks was found to lead to retest effects when considering RTs as outcome measures. Effect sizes of a quarter *SD* for the first to second test, more than a third *SD* for the first to third test and almost half a *SD* for the first to fourth test were observed. Note that effects have to be interpreted as RT decrease, meaning the more negative the effect size, the higher the retest effect. These retest effects are comparably large as retest effects considering score gains [2,5,6,7] and the finding is in line with most primary studies (e.g., [40]). RT reduction due to retesting can be explained by the three groups of causes put forward by Lievens et al. (2007) [11] that might apply to RT reduction as well as to score gains. Also, the automatization of mental processes that takes place when retested can account for RT reductions when mental speed tests are administered multiple times [44,46]. In addition, automatization and the three causes might reciprocally support each other.

As expected by theoretical assumptions according to the power law of practice [48,49,50] and automatization processes [44,45,46,47], the gain from the first to second test was largest. Interestingly, the RT reductions between second and third test did not differ from those between the third and fourth test, suggesting further RT reductions after the third test. A plateau does not seem to be reached after four test administrations and further acceleration of mental processing might take place. This is of special interest, as many studies with cognitive ability tests focusing on score gains and their course over multiple test repetitions have found retest effects to reach a plateau somewhat earlier [7,107,108,109,110]. Thus, retest effects might be interpreted differently when RT is the outcome measure than when the total score is. The latter statement is in line with Ackerman (1987) [34], as mean and variability are not always preserved when transforming RT to score gains. Other studies are in line with this finding and showed dissimilar growth of attentional speed and accuracy during the course of testing; in the research of Goldhammer et al. (2010) [35], these two variables followed linear and logarithmic trajectories respectively. However, it has to be considered that only few studies administered more than two tests and that the average sample size for effects of multiple test administrations was low. This challenges the generalizability of results for multiple retests and clearly calls for future research, as discussed below.

Only a low proportion of eligible studies used alternate test forms of mental speed tests, which is why it was impossible to test H2 and H4a. As it seems a stable result that alternate test forms show smaller retest effects compared to identical test forms [2,7,21,24], a replication of this finding for the case of RT outcomes and mental speed tasks would have been of interest. A possible explanation for why most studies used identical test forms for retesting can be derived when taking into account that the use of alternate test forms is one of the most commonly recommended methods to prevent retest effects [7,21,24]. Retest effects have only rarely been issued neither for mental speed nor for RT outcomes and this might have led to a low awareness of retest effects for these kind of tests and outcomes and a low motivation to prevent them, resulting in a rare use of alternate test forms.

Task complexity moderated the size of RT reductions due to retesting: Smaller effects were found for simple compared to more complex mental speed tasks. This can possibly be explained by a broader reduction of construct-irrelevant factors, such as test anxiety or rule incomprehension and also by an increase in test-specific strategies when working repeatedly on more complex mental speed tasks. In simple mental speed tasks, a testee simply has to react fast to a stimulus, which does not allow any strategy use. In a complex task, such as a TMT (Part B), strategies might apply and foster performance. Also, automatization can be argued to take place faster in consistent tasks [Ackerman], which might explain why smaller effects were found for simple tasks and also why RT reductions in simple tasks seemed to show no further development after the second test. In fact, when comparing first to third and second to third test, retesting with simple mental speed tasks did not result in a significant retest effect and a plateau seemed to be reached after the second test administration. For easy mental speed tasks, automatization seems to be completed after the second test administration. On the contrary, complex tasks showed larger RT reductions and increasing retest effects over test repetitions and a plateau does not seem to be reached. Automatization actually seems to take longer for more complex tasks and also construct-irrelevant factors and test-specific strategies might still play role when retested a few times.

The test-retest interval between tests seems to play a role in RT reductions between the first and second test administration. According to our results, an RT reduction of a quarter *SD* is expected when persons are retested immediately or after a short term. Furthermore, our results show that retest effects are no longer evidenced when approximately five years pass from the first to second test administration. This is in line with previous research findings of test-retest interval moderating the size of retest effects regarding score gains [2,5,7,64]. A longer time interval between test administrations would imply that fewer contaminating factors influence RTs, as individuals are less prone to remember the items or to gain knowledge about test-specific response strategies, thus leading to less automatization of mental processes. Though, this effect was small and, for further test repetitions, the length of the test-retest interval did not moderate RT reduction effect sizes. Note that the test-retest interval for studies with three or four test administrations was smaller than for all studies taken together (see Table 1). This might have led to a restricted variance in the test-retest intervals given for three and four test administrations. However, the time interval between test administrations might not produce the same effect on the size of RT reduction as it does on score gains. Assumed that automatization remains stable over long test-retest intervals and that automatization processes proceed implicitly, no explicit memory of the tests, which decreases with the test-retest interval, might be necessary in order for mechanisms to accelerate.

Age moderated the size of RT reduction effects only when comparing RTs from the third and fourth test, such as RT reductions became smaller with age. Age has been found to have a negative influence on the size of score gain retest effects by, e.g., Calamia et al. (2012) [6], Lo, Humphreys, Byrne and Pachana (2012) [111], Schleicher, Van Iddekinge, Morgeson and Campion (2010) [112] and Van Iddekinge et al. (2011) [66]. This could be explained by a decreasing fluid intelligence with age [67], a lower ability to maintain and update information [68] and a resulting decreasing ability to learn from prior test experiences [66]. For other comparisons between test administrations, however, no moderating effect of age was found. Verhaeghen (2015) [4] and Baltes et al. (1986) [40] also reported that age did not moderate retest effect sizes. As we restricted participant age to 12 to 70 years, this might limit the extent to which age has an influence on RT reductions in our data. Indeed, Strobach & Schubert (2017) [113] argue that there are no age differences in the automatization ability, thus contradicting Maquestiaux et al. [69,70]. Within the selected age groups, there might be no substantial differences in fluid intelligence and automatization ability, thereby leading to mostly equally large RT reduction effects for all participants included in the analysis. Also, cohort effects have to be considered as confounded with age. As eligible studies have been published between 2010 and 2016, results regarding age might be influenced by characteristics of the observed cohorts and might not be generalizable. Note that age is often used as an approximation of another underlying variable, as, in this case, of fluid intelligence. Testing variables such as fluid intelligence or learning ability directly might lead to results that are more informative and the possibility of a narrow interpretation of mechanisms actually causing retest effects. Meta-analysis, however, is limited to using approximating variables that are reported by eligible studies most often.

### 4.2. Limitations

When inspecting the effect sizes and their variance in detail, the high heterogeneity of effects becomes evident. A *SD* of the true effect of τ = 0.24 for the effect from first to second test leads to a prediction interval of [−0.73, 0.26], which stresses the high heterogeneity of effects. In fact, some studies report an increase of RT when retested, although this increase was mostly insignificant from zero. Redick (2015) [114] indeed found a few control groups of working memory trainings, who proceed a similar simple retesting procedure, to become worse in a criterion task when retested. Scharfen et al. (in press) [7] have also reported a high heterogeneity of the effect and lower scores in a retest. A higher RT in a second compared to a first test, can possibly be explained by fluctuations in motivation, or fatigue. Moderators could only partly explain differences between effect sizes, with task complexity being a promising determinant of the size of RT reductions. On the one hand, the size of τ should be expected due to the heterogeneity of studies about the present topic. On the other hand, it means that there might be additional moderating variables that account for differences between effect sizes. Regarding score gains, Randall & Villado (2017) [8] give a broad theoretical framework and suggestions for possible moderating variables explaining differences between retest effects regarding score gains. For RT reduction, similar moderators might be plausible, as similar mechanisms are assumed accountable for RT reductions when retested. Variables such as motivation, feedback on the correctness of the given answer and general mental ability of the sample might be of interest in this regard. This meta-analytic study is limited to the information given by eligible studies and to the existence of eligible studies that have been conducted so far. Unfortunately, only very few studies used alternate test forms of a mental speed task, so it was impossible to test this as a moderator of RT reductions. It might be problematic for future meta-analyses and reviews on the topic to gather information about the variables referred to above (feedback, motivation and general mental ability) as well, as information on them is rarely reported. Also, only few studies administer more than four test administrations, which is why no further RT reduction effects could be analyzed beyond the fourth test administration and results for multiple retests have to be interpreted carefully.

Based on the results of this meta-analysis, it was not possible to conclude about which of the causes of retest effects might hold to what degree. Variables linked directly to the causes, such as test anxiety, motivation or strategy use, were reported in only a very low number of studies. This suggests that more research is needed investigating possible moderating variables directly to fully understand retest effects and mechanisms causing them, as also claimed by Randall & Villado (2017) [8].

Lastly, this meta-analysis did not control for statistical artifacts such as unreliability, restriction of range, or construct invalidity, as suggested by Hunter and Schmidt (1990) [115]. Although RT measures should have of a low measurement error and thus a high reliability, statistical artifacts might have an influence on the results.

### 4.3. Future Research

As only few studies were found that performed multiple test administration, future research should focus on multiple retests and especially analyze mechanisms leading to retest effects over several repetitions in more detail. Although a few studies have investigated to what degree the proposed causes of retest effects apply [20,22,25], for multiple retesting, these mechanisms and their changes when tests are administered multiple times have not been understood in detail.

A systematic review of test-retest correlations between RTs from several repetitions would contribute to the literature, as also for correlations, meta-analytic work has been focusing on score gains (e.g., [95]). For the present analysis, test-retest correlations were only rarely reported by eligible studies. A review explicitly focusing test-retest correlations might include a different sample of studies that might result in a representative estimation of this correlation.

More research is needed to understand the mechanisms that cause RT reduction effects, as retesting research has been mainly concentrating on score gains. The automatization of mental processes seems to play a crucial role in this regard [41,42,43,44,45,46,47], whereas deliberations from the field of score gains might be relevant as well [11], with mechanisms supposedly supporting each other. A comparison of effects sizes between RT reductions and score gains due to retesting would be of special interest, as the present meta-analytic review indicates that RT reduction effects might follow different patterns over multiple repetitions compared to score gains.

This study has focused on mental speed tasks. For this kind of cognitive ability tasks, outcomes are mostly reported as RTs. Though, it might be interesting to investigate the role of RT reduction in other cognitive domains as well, as differences between cognitive operations have been observed in score gains due to retesting [63,108,110]. In this regard, *g* loadings might explain differences in RT reduction. *g* loadings differ between cognitive domains and have been shown to predict the size of retest effects, such as tasks with higher *g* loading show smaller retest effects [19]. Randall & Villado 2017 [8] argue that *g* loaded tasks are much harder to deter. For the present meta-analysis, we observe a restricted range of *g* loadings because we focus on mental speed tasks only, which are homogeneous in their measured ability by definition. Within mental speed tests, such a differentiation thus seemed challenging because of the restricted range of *g* loadings in the observed mental speed tasks. For more highly *g* loaded tasks than those measuring mental speed, smaller RT reductions than those found by this analysis would be expected.

Moreover, RT reductions might be relevant when retesting with other constructs than cognitive ability as well, such as personality, e.g., Hausknecht (2010) [116], has investigated retest effects in personality tests and reported large effects regarding scores on personality dimensions. These and related results stress the possibility of faking when retested [39,116,117]. It is reasonable to assume that retest effects regarding RTs would also be observed in personality tests, although RTs might be even more rarely reported compared to cognitive ability retesting. However, if RT retest effects are comparably large as those observed in scores, RTs might give hints on which testees might be retaking a personality assessment.

Relevant to moderator analyses, studies that retest multiple times in a broad range of test-retest intervals might give further insight into its moderating influence on the size of the effect. More studies are needed that use large test-retest intervals and mental speed tasks with a low complexity for a third or fourth test. In addition, as it was not possible to test H2 and H4 because only few studies used alternate test forms, the role of equivalence of test forms should be investigated for mental speed tasks and RT outcomes. If the finding that alternate test forms show smaller retest effects than identical ones, the use of alternate test forms could be an effortless method to prevent retest effects in these kinds of tests as well.

The results of this meta-analytic study only partly support the assumption that age moderates the size of RT reduction retest effects. As we restricted the participants’ age to 12 to 70 years, different results might be expected when focusing on children or elderly people. As cognitive development at these stages of life is not as stable as between 12 and 70 years, different effects might be observed that give further insight in how age and retest effects are related.

Several methods have been reported to control for retest effects regarding score gains [21,24]. However, for RT outcomes these might not apply. Research should thus be seeking effective mechanisms controlling for RT retest effects in applied settings.

### 4.4. Practical Implications

When mental speed tests are used in applied settings, retest effects have to be considered by practitioners when diagnoses are based on RT as well as on score outcomes. This is especially important when tests are administered multiple times or testees are very familiar with the test criterion. The results of this meta-analytic review show that RT outcomes are as prone to retest effects as scores and that they might even take longer to reach a learning plateau. To illustrate, in the study of Collie et al. (2003) [53], it took participants on average 294 ms less to answer during retest (Simple Reaction Time) compared to the first test. When taking the test for a third time, participants were again 14 ms faster compared to the second test and, when taking it for a fourth time, they were on average faster by 14 ms compared to the third test. In order to be able to derive a diagnosis from a test result, it is critical to understand which of these results might be the most reliable and valid. With regard to score gains, results are mixed concerning which test is the most valid, whether the initial test or the retest [17,18,19]. For RT outcomes, this question arises as well.

For the two most common mental speed tests, this meta-analytic study gives estimates of how RT changes due to retesting. For the TMT, RTs are reduced on average by a third *SD* for a second test and by almost half a *SD* for a third test. For the Stroop test, the effects were slightly smaller, with RT reductions of a fifth *SD* from first to second and two fifth *SDs* from first to third test. Note that RT reductions between the first and second test were as equally large as those between second and third test, suggesting different patterns of RT reductions in Stroop tests compared to other mental speed tasks. Practitioners using these tests are encouraged to use these estimates of RT reduction effects due to retesting when tests are administered multiple times to the same person and inferences are drawn from their RT results.

It is an important finding that RT reductions in simple mental speed tasks were smaller compared to more complex kinds of tasks and that RT reduction due to retesting with simple tasks seemed to stagnate after the second test. Using simple mental speed tasks repeatedly might thus not be affected by retest effect as much as other kinds of tasks and outcomes. If retest effects are to be prevented, e.g., in longitudinal research on cognitive development, experimental evaluations, clinical diagnosis or aptitude assessments, the use of simple mental speed tasks and RT outcomes can thus be recommended.

Generally, for the use of mental speed tasks in applied settings, it cannot be endorsed to interpret RT outcomes from repeated administrations without concerns. It is rather suggested to use unique or very simple tasks, not let participants prepare themselves in advance and not use the same tests multiple times for the same persons. It can also be a great help in interpreting the effects to ask the applicant about his familiarity with the task [21] to be able to more reliably interpret their results.

## Figures and Tables

**Table 1:** Study, Sample and Test Characteristics.

No. of Administrations	Level	Characteristic	*M*	*SD*	Mdn	Min	Max	% NA
2	Study (*m* = 36)	year of publication	2010.00	4.13	2012.00	1999.00	2016.00	0.00
		no. of administrations	3.028	1.96	2.00	2.00	12.00	0.00
		TR interval (weeks)	32.89	75.32	3.00	0.00	312.90	0.00
		% control groups	36.73					0.00
	Sample (*k* = 49)	*N*	445.10	2755.30	25.00	9.00	19,330	0.00
		age	51.70	19.74	54.50	14.8	68.80	0.00
		% male	41.31	17.65	45.50	0.00	100.00	12.25
	Test (*o* = 128)	% alternate test forms	3.18					1.56
		*SMCR*_1.2_	−0.22	0.28	−0.19	−1.19	0.46	0.00
		*SE(SMCR*_1.2_)	0.17	0.06	0.16	0.01	0.31	0.00
3	Study (*m* = 14)	year of publication	2008.00	4.05	2008.00	1999.00	2014.00	0.00
		no. of administrations	4.64	2.40	4.00	3.00	12.00	0.00
		TR interval (weeks)	4.53	5.84	2.14	0.00	17.43	0.00
		% control groups	12.00					0.00
	Sample (*k* = 25)	*N*	34.36	22.67	25.00	10.00	113.00	0.00
		age	31.16	17.21	22.20	15.41	67.35	0.00
		% male	39.63	19.37	33.63	0.00	100.00	12.00
	Test (*o* = 58)	% alternate test forms	3.57					3.44
		*SMCR*_1.3_	−0.40	0.48	−0.27	−1.85	0.35	0.00
		*SE(SMCR*_1.3_)	0.17	0.06	0.16	0.08	0.34	0.00
4	Study (*m* = 9)	year of publication	2007.00	4.11	2007.00	1999.00	2013.00	0.00
		no. of administrations	5.56	2.60	5.00	4.00	12.00	0.00
		TR interval (weeks)	4.38	6.71	0.51	0.00	20.00	0.00
		% control groups	14.29					0.00
	Sample (*k* = 14)	*N*	34.36	27.79	24.00	10.00	113.00	0.00
		age	34.97	18.58	23.00	15.41	63.68	0.00
		% male	40.58	13.20	41.18	0.00	60.00	7.14
	Test (*o* = 26)	% alternate test forms	8.33					7.69
		*SMCR*_1.4_	−0.57	0.50	−0.55	−1.85	0.33	0.00
		*SE(SMCR*_1.4_)	0.17	0.08	0.16	0.08	0.39	0.00

Note. *SE(SMCR)* = $\sqrt{Var\left(SMCR\right)}$.

**Table 2:** Meta-Analytically Derived Effect Sizes for RT Reduction due to Retesting.

Comparison	*m*	*k*	*o*	*N*	*SMCR*	*SE*	95% CI	*p*	τ
1.2	36	49	128	21,810	−0.237	0.040	[−0.318, −0.155]	<0.001	0.238
1.3	14	25	58	859	−0.367	0.075	[−0.519, −0.215]	<0.001	0.399
1.4	9	14	26	481	−0.499	0.082	[−0.666, −0.333]	<0.001	0.424
2.3					−0.131	0.054	[−0.241, −0.021]	0.021	
3.4					−0.132	0.026	[−0.186, −0.079]	<0.001	
1.2 vs. 2.3					−0.106	0.059	[−0.226, 0.014]	0.041	
2.3 vs. 3.4					0.002	0.055	[−0.111, 0.114]	0.979	

Note. *m* = number of studies, *k* = number of samples, *o* = number of outcomes, *N* = total sample size, *SMCR* = standardized mean change with raw score standardization, *SE* = standard error, CI = confidence interval, τ = estimated overall *SD* of the true effect.

**Table 3:** Meta-Analytically Derived Effect Sizes for RT Reduction Due to Retesting: Stroop tasks and variants of the TMT.

Test	Comparison	*m*	*k*	*o*	*N*	*SMCR*	*SE*	95% CI	*p*
TMT	1.2	11	14	28	445	−0.331	0.063	[−0.460, −0.201]	<0.001
	1.3	4	7	11	225	−0.448	0.120	[−0.693, −0.203]	<0.001
	2.3					−0.117	0.065	[−0.250, 0.016]	0.082
	1.2 vs. 2.3					−0.214	0.046	[−0.307, −0.120]	<0.001
Stroop	1.2	7	8	15	211	−0.211	0.065	[−0.344, −0.078]	0.002
	1.3	2	2	7	75	−0.399	0.075	[−0.552, −0.247]	<0.001
	2.3					−0.189	0.017	[−0.223, −0.154]	<0.001
	1.2 vs. 2.3					−0.022	0.059	[−0.142, 0.098]	0.352

Note. *m* = number of studies, *k* = number of samples, *o* = number of outcomes, *N* = total sample size, *SMCR* = standardized mean change with raw score standardization, *SE* = standard error, CI = confidence interval, TMT = Trail Making Test.

**Table 4:** Subgroup Analysis for Task Complexity.

Comparison	Complexity	*o*	*SMCR*	*SE*	95% CI	*p*	∆*SMCR*_simple-complex_	*p* (∆*SMCR*_simple-complex_)	τ	∆_τ_^2^
1.2	simple	25	−0.108	0.044	[−0−199, 0.018]	0.021	0.159	0.001	0.224	0.117
	complex	103	−0.268	0.044	[−0.357, −0.179]	<0.001				
1.3	simple	12	−0.130	0.070	[−0.272, 0.012]	0.071	0.295	0.004	0.386	0.063
	complex	46	−0.425	0.091	[−0.611, −0.239]	<0.001				
2.3	simple		−0.022	0.047	[−0.117, 0.074]	0.645	0.135	0.029		
	complex		−0.157	0.068	[−0.296, −0.018]	0.028				
1.2 vs. 2.3	simple		−0.087	0.059	[−0.207, 0.034]	0.152	0.024	0.347		
	complex		−0.157	0.068	[−0.296, −0.018]	0.122				

Note. *o* = number of outcomes, *SMCR* = standardized mean change with raw score standardization, *SE* = standard error, CI = confidence interval, ∆*SMCR*_simple-complex_ = difference of RT reduction between complex and simple tasks, τ = estimated overall *SD* of the true effect from the model including the moderator, ∆**_τ_**^2^ = proportion of explained variance of the presented model (model 2) compared to the model without moderators (model 1), as $\frac{\tau_{model1}^{2}-\tau_{model2}^{2}}{\tau_{model1}^{2}}$.

**Table 5:** Meta-Regressions for Test-Retest Interval and Participant Age.

Moderator	Comparison	Coefficient	*SMCR*	*SE*	95% CI	*p*	τ	∆_τ_^2^
Test-Retest Interval (weeks)	1.2	Int	−0.258	0.046	[−0.353, −0.164]	<0.001	0.235	0.026
		*b*	0.001	0.001	[−0.000, 0.002]	0.038		
	1.3	Int	−0.414	0.096	[−0.611, −0.217]	0.001	0.399	0.000
		*b*	0.007	0.008	[−0.010, 0.024]	0.198		
	1.4	Int	−0.543	0.106	[−0.759, −0.327]	<0.001	0.425	0.000
		*b*	0.004	0.007	[−0.010, 0.017]	0.280		
	2.3	Int	−0.156	0.078	[−0.316, 0.004]	0.056		
		*b*	0.006	0.008	[−0.011, 0.024]	0.224		
	3.4	Int	−0.129	0.028	[−0.187, −0.071]	<0.001		
		*b*	−0.003	0.004	[−0.011, 0.004]	0.384		
Age (yrs)	1.2	Int	−0.220	0.861	[−0.396, −0.044]	0.016	0.241	0.000
		*b*	−0.001	0.002	[−0.631, −0.021]	0.803		
	1.3	Int	−0.326	0.149	[−0.631, −0.021]	0.037	0.401	0.000
		*b*	−0.001	0.003	[−0.005, 0.004]	0.717		
	1.4	Int	−0.553	0.176	[−0.912, −0.195]	0.004	0.426	0.000
		*b*	0.001	0.004	[−0.006, 0.008]	0.392		
	2.3	Int	−0.106	0.103	[−0.317, 0.105]	0.312		
		*b*	−0.001	0.002	[−0.004, 0.003]	0.723		
	3.4	Int	−0.227	0.043	[−0.314, −0.141]	<0.001		
		*b*	0.002	0.007	[0.006, 0.004]	0.004		

Note. *SMCR* = standardized mean change with raw score standardization, *SE* = standard error, CI = confidence interval, τ = estimated overall *SD* of the true effect from the model including moderators, ∆_τ_^2^ = proportion of explained variance of the presented model (model 2) compared to the model without moderators (model 1), as $\frac{\tau_{model1}^{2}-\tau_{model2}^{2}}{\tau_{model1}^{2}}$ Int = Intercept, *b* = regression weight.

## Supplementary Materials

The following are available online at http://www.mdpi.com/2079-3200/6/1/6/s1, Figure S1: Forest Plot, File S1: data.rda, File S2: script.R.

## Appendix A

**Table A1:** List of Eligible Studies, their Main Characteristics and Effect Sizes.

Study	Goal of Study	Sample No.	*n*	Age (*M*)	TR Interval (Weeks)	Test	Subtest	Complexity	*SMCR*		
									1.2	1.3	1.4
1. Anastasopoulou et al., 1999 [118]	retest effects	1	17	21.3	0.00, 0.03, 0.03	Response Time Task		complex	−0.54	−0.66	−1.12
		2	17	21.3	0.00, 0.03, 0.03	Response Time Task		complex	−0.24	−0.21	−0.36
2. Baird et al., 2007 [119]	retest effects	3	13	17.9	0.00	Computerized Test of Information Processing	choice RT	complex	0.30	0.07	
					0.00	Computerized Test of Information Processing	simple RT	simple	0.04	−0.29	
					0.00	Computerized Test of Information Processing	semantic RT	complex	−0.35	−0.73	
		4	13	19.5	1.00	Computerized Test of Information Processing	choice RT	complex	0.07	0.35	
					1.00	Computerized Test of Information Processing	simple RT	simple	−0.04	0.02	
					1.00	Computerized Test of Information Processing	semantic RT	complex	−0.25	−0.14	
		5	13	20.5	12.86	Computerized Test of Information Processing	choice RT	complex	0.00		
					12.86	Computerized Test of Information Processing	simple RT	simple	−0.24		
					12.86	Computerized Test of Information Processing	semantic RT	complex	−0.37		
3. Baniqued et al., 2014 [120]	intervention: passive CG	6	61	20.7	2.50	Attention Network Test	no cue trials	simple	0.16		
					2.50	Attention Network Test	incongruent-congruent trials	complex	−0.29		
					2.50	Attention Network Test	location trials	complex	0.03		
					2.50	Stroop Test	incongruent-neutral trials	simple	0.00		
					2.50	Stroop Test	incongruent-congruent trials	complex	−0.01		
					2.50	Trail Making Test	numbers	complex	0.25		
					2.50	Trail Making Test	letters	complex	−0.57		
					2.50	Trail Making Test	numbers and letters	complex	−0.19		
4. Bartels et al., 2010 [107]	retest effects	7	36	47.3	2.29, 6.00, 9.00	Attention Test Battery	alterness	simple	−0.07	−0.09	−0.15
					2.29, 6.00, 9.00	Attention Test Battery	visual scanning	simple	−0.58	−0.80	−1.00
					2.29, 6.00, 9.00	Trail Making Test	part B	complex	−0.30	−0.36	−0.60
					2.29, 6.00, 9.00	Trail Making Test	part A	complex	−0.40	−0.44	−0.59
5. Buck et al., 2008 [121]	retest effects	8	48	20	1.00, 2.00	Trail Making Test	part A	complex	−0.09	−0.13	
					1.00, 2.00	Trail Making Test	part B	complex	−0.13	−0.19	
		9	44	20	1.00, 2.00	Delis-Kaplan Trail Making Test	numbers task	complex	−0.13	−0.16	
					1.00, 2.00	Delis-Kaplan Trail Making Test	letters task	complex	−0.04	−0.11	
					1.00, 2.00	Delis-Kaplan Trail Making Test	number-letter task	complex	−0.11	−0.13	
		10	42	20	1.00, 2.00	Comprehensive Trail Making Test	trial 1	complex	−0.10	−0.19	
					1.00, 2.00	Comprehensive Trail Making Test	trial 5	complex	−0.07	−0.12	
		11	48	20	1.00, 2.00	Planned Connections	trial 6	complex	−0.07	−0.09	
					1.00, 2.00	Planned Connections	trial 8	complex	−0.07	−0.10	
6. Bühner et al., 2006 [52]	retest effects	12	25	22.2	0.00, 0.00, 0.00	Attention Test Battery	audio	complex	0.09	0.00	−0.14
		13	23	22.2	0.00, 0.00, 0.00	Attention Test Battery	squares	complex	−0.08	−0.61	−0.62
		14	24	22.2	0.00, 0.00, 0.00	Attention Test Battery	squares and audio	complex	0.05	−0.06	−0.34
		15	24	22.2	0.00, 0.00, 0.00	Attention Test Battery	Go/Nogo	complex	−0.05	−0.35	−0.64
7. Bürki et al., 2014 [3]	intervention: passive CG	16	21	25.52	3.00	letter comparison task		complex	0.08		
					3.00	simple reaction time task		simple	0.07		
					3.00	pattern comparison task		complex	−0.06		
8. Collie et al., 2003 [53]	retest effects	17	113	63.68	0.03, 0.09, 0.18	simple reaction time test		simple	−0.15	−0.26	−0.57
					0.18, 0.71, 0.89	choice reaction time test		complex	−0.30	−0.47	−0.52
					0.18, 0.71, 0.89	complex reaction time test		complex	−0.34	−0.44	−0.51
					0.18, 0.71, 0.89	continuous performance test		simple	−0.29	−0.34	−0.39
					0.21, 0.71, 0.89	matching test		complex	−0.42	−0.45	−0.39
9. Colom et al., 2013 [122]	retest effects	18	28	18.2	21.43	odd-even Flanker task		complex	−0.17		
					21.43	right-left Simon task		complex	0.18		
					21.43	vowel-consonant Flanker task		complex	−0.20		
10. Dingwall et al., 2009 [123]	test-retest reliability	19	17	15.41	2.14, 4.29, 6.29	CogState Battery	identification	simple	0.32	0.32	0.21
					2.14, 4.29, 6.29	CogState Battery	detection	simple	0.06	0.19	0.00
11. Dolan et al., 2013 [124]	test-retest reliability	20	9	22	0.29	choice reaction time test		complex	−0.35		
					0.29	rapid visual information processing test		simple	0.40		
					0.29	simple reaction time test		simple	−0.26		
					0.29	Stroop test	baseline trials	simple	−0.35		
					0.29	Stroop test	interference trials	complex	−0.27		
12. Elbin et al., 2011 [125]	test-retest reliability	21	369	14.8	62.57	ImPACT	reaction time subscale	complex	−0.37		
13. Enge et al., 2014 [126]	intervention: passive CG	22	38	21.3	3.00, 17.43	Go/No-go task		complex	−1.19	−1.68	
					3.00, 17.43	Stop Signal		complex	−0.78	−0.80	
					3.00, 17.43	Stroop test		complex	−0.63	−0.74	
14. Falleti et al., 2006 [127]	retest effects	23	45	21.64	0.00, 0.00, 0.00	CogState Battery	choice reaction time	complex	0.16	0.33	0.33
					0.00, 0.00, 0.00	CogState Battery	simple reaction time	simple	0.00	0.00	−0.16
					0.00, 0.00, 0.00	CogState Battery	complex reaction time	complex	0.00	0.00	−0.22
					0.00, 0.00, 0.00	CogState Battery	matching	complex	−0.59	−0.69	−0.79
		24	55	32.69	0.00, 4.29	CogState Battery	choice reaction time	complex	0.00	0.18	
					0.00, 4.29	CogState Battery	continuous associate learning	complex	−0.06	−0.06	
					0.00, 4.29	CogState Battery	complex reaction time	complex	−0.12	−0.12	
					0.00, 4.29	CogState Battery	simple reaction time	simple	−0.20	−0.10	
15. Gil-Gouveia et al., 2016 [128]	longitudinal change in migraineurs: healthy CG	25	24	33.3	6.43	Trail Making Test	part A	complex	−0.37		
					6.43	Trail Making Test	part B	complex	−0.35		
16. Hagemeister, 2007 [1]	retest effects	26	60	28	0.01, 0.50, 0.51	attention task		complex	−0.48	−1.41	−1.44
		27	59	23	0.01, 0.50, 0.51	attention task		complex	−0.46	−1.85	−1.85
17. Iuliano et al., 2015 [129]	intervention: passive CG	28	20	66.47	12.00	Attentive Matrices Test		complex	−0.09		
					12.00	Stroop test		complex	0.18		
					12.00	Trail Making Test	part A	complex	−0.16		
					12.00	Trail Making Test	part B	complex	0.05		
18. Langenecker et al., 2007 [130]	test-retest reliability	29	28	18.9	3.00	Go/No-go task	level 1	complex	−0.33		
					3.00	Trail Making Test		complex	−0.58		
					3.00	Trail Making Test		complex	−0.73		
19. Lemay et al., 2004 [131]	retest effects, test-retest reliability	30	37	67.35	2.00, 4.00	Stroop test	reading subtest	simple	−0.06	−0.06	
					2.00, 4.00	Stroop test	naming subtest	simple	−0.30	−0.41	
					2.00, 4.00	Stroop test	interference subtest	complex	−0.64	−0.90	
					2.00, 4.00	Stroop test	flexibility subtest	complex	−0.54	−0.81	
					2.00, 4.00	Stroop test	inter-naming subtest	complex	−0.64	−0.92	
					2.00, 4.00	Stroop test	flex-naming subtest	complex	−0.51	−0.78	
20. Levine et al., 2004 [132]	retest effects	31	605	40.2	35.86	California Computerized Assessment Package	simple reaction time	simple	−0.13		
					35.86	California Computerized Assessment Package	choice reaction time trials 1	complex	0.03		
					35.86	California Computerized Assessment Package	choice traction time trials 2	complex	−0.11		
21. Lyall et al., 2016 [102]	longitudinal change	32	19327	54.5	225.78	reaction time test		simple	0.08		
22. Mehlsen et al., 2008 [133]	longitudinal change in breast cancer patients: healthy CG	33	17	39.3	14.00	Trail Making Test	part A	complex	−0.62		
					14.00	Trail Making Test	part B	complex	−0.59		
23. Mora et al., 2013 [134]	longitudinal change in bipolar patients: healthy CG	34	26	41.38	312.86	Continuous Performance Test II	hit reaction time	complex	0.03		
					312.86	Trail Making Test B	part B	complex	−0.01		
24. Oelhafen et al., 2013 [135]	intervention: passive CG	35	16	25.2	21.00	Attention Network Test congruent	congruent trials	simple	−0.19		
					21.00	Attention Network Test incongruent	incongruent trials	complex	−0.42		
25. Ownby et al., 2016 [136]	retest effects	36	51	28.92	55.33	Colored Trails	test 1	complex	−0.36		
					55.33	Colored Trails	test 2	complex	−0.25		
26. Register-Mihalik et al., 2012 [137]	test-retest-reliability	37	20	16	0.26, 0.49	Trail Making Test	part B	complex	−0.98	−1.48	
					0.26, 0.49	ImPACT	reaction time subscale	complex	−0.48	−0.32	
		38	20	20	0.26, 0.49	Trail Making Test	part B	complex	−0.62	−1.08	
					0.26, 0.49	ImPACT	reaction time subscale	complex	−0.16	−0.16	
27. Richmond et al., 2014 [138]	intervention: passive CG	39	18	21.6	2.29	psychomotor vigilance task		simple	0.46		
					2.29	sustained attention response task		simple	−0.50		
					2.29	Stroop test		complex	−0.19		
28. Salminen et al., 2012 [139]	intervention: passive CG	40	18	24.5	3.00	auditory discrimination task	SOA 100	complex	−0.40		
					3.00	auditory discrimination task	SOA 400	complex	−0.24		
					3.00	auditory discrimination task	SOA 50	complex	−0.34		
					3.00	visual discrimination task	SOA 100	complex	−0.59		
					3.00	visual discrimination task	SOA 400	complex	−0.57		
					3.00	visual discrimination task	SOA 50	complex	−0.57		
					3.00	task switching	repetition trials	complex	−0.47		
					3.00	task switching	single-task trials	complex	−0.36		
29. Sandberg et al., 2014 [140]	intervention: passive CG	41	13	24.62	5.00	Flanker task		complex	−0.06		
					5.00	Stroop test		complex	−0.16		
		42	15	68.8	5.00	Flanker task		complex	−0.32		
					5.00	Stroop test		complex	−0.10		
30. Schatz, 2010 [141]	test-retest-reliability	43	95	18.8	104.29	ImPACT	reaction time subscale	complex	−0.17		
31. Schmidt et al., 2013 [142]	intervention: passive CG	44	11	33.8	6.00, 13.50, 20.00	Trail Making Test A	part A	complex	−0.39	−0.40	−0.59
32. Schranz & Osterode, 2009 [143]	retest effects	45	10	42.13	1.141 2,29, 3.43	Determination Test	Action	complex	−0.52	−1.02	−1.29
					1.141 2,29, 3.43	Determination Test	Reaction	complex	−0.56	−0.91	−1.10
33. Sharma et al., 2013 [144]	intervention: passive CG	46	28	19	12.00	auditory reaction time test		complex	0.01		
					12.00	Letter Cancellation Test		complex	−0.10		
					12.00	Trail Making Test A	part A	complex	−0.17		
					12.00	Trail Making Test B	part B	complex	−0.23		
					12.00	visual reaction time test		complex	0.02		
					12.00	visual reaction time test		complex	0.00		
34. Soveri et al., 2013 [145]	intervention: passive CG	47	14	23.14	2.50	Simon task	congruent trials	simple	0.14		
					2.50	Simon task	incongruent trials	complex	−0.12		
35. Steinborn et al., 2008 [146]	retest effects	48	89	24.5	0.43	Serial Mental Addition and Comparison Task		complex	−0.74		
36. Weglage et al., 2013 [147]	longitudinal change in clinical sample: healthy CG	49	46	34.2	260.71	Connecting Numbers		complex	−0.30		

Note. CG = control group, ImPACT = Immediate Post-Concussion Assessment and Cognitive Test.
